# Using i-PARIHS to assess implementation of the Surgical Safety Checklist: an international qualitative study

**DOI:** 10.1186/s12913-022-08680-1

**Published:** 2022-10-25

**Authors:** Meagan E. Elam, Christopher J. Louis, Mary E. Brindle, Jonathan Woodson, Jacey A. Greece

**Affiliations:** 1 Boston University School of Public Health, 715 Albany St, Boston, MA 02118 USA; 2 Ariadne Labs, 401 Park Dr 3rd Floor, Boston, MA 02215 USA; 3 University of Calgary, 2500 University Dr NW, Calgary, AB T2N 1N4 Canada; 4 Boston University School of Medicine, 72 E Concord St, Boston, MA 02118 USA

**Keywords:** Surgical safety checklist, i-PARIHS framework, Implementation strategies, Quality and safety

## Abstract

**Background:**

Strategies selected to implement the WHO’s Surgical Safety Checklist (SSC) are key factors in its ability to improve patient safety. Underutilization of implementation frameworks for informing implementation processes hinders our understanding of the checklists’ varying effectiveness in different contexts. This study explored the extent to which SSC implementation practices could be assessed through the i-PARIHS framework and examined how it could support development of targeted recommendations to improve SSC implementation in high-income settings.

**Methods:**

This qualitative study utilized interviews with surgical team members and health administrators from five high-income countries to understand the key elements necessary for successful implementation of the SSC. Using thematic analysis, we identified within and across-case themes that were mapped to the i-PARIHS framework constructs. Gaps in current implementation strategies were identified, and the utility of i-PARIHS to guide future efforts was assessed.

**Results:**

Fifty-one multi-disciplinary clinicians and health administrators completed interviews. We identified themes that impacted SSC implementation in each of the four i-PARIHS constructs and several that spanned multiple constructs. Within innovation, a disconnect between the clinical outcomes-focused evidence in the literature and interviewees’ patient-safety focus on observable results reduced the SSC’s perceived relevance. Within recipients, existing surgical team hierarchies impacted checklist engagement, but this could be addressed through a shared leadership model. Within context, organizational priorities resulting in time pressures on surgical teams were at odds with SSC patient safety goals and reduced fidelity. At a health system level, employing surgical team members through the state or health region resulted in significant challenges in enforcing checklist use in private vs public hospitals. Within its facilitation construct, i-PARIHS includes limited definitions of facilitation processes. We identified using multiple interdisciplinary champions; establishing checklist performance feedback mechanisms; and modifying checklist processes, such as implementing a full-team huddle, as facilitators of successful SSC implementation.

**Conclusion:**

The i-PARIHS framework enabled a comprehensive assessment of current implementation strategies, identifying key gaps and allowed for recommending targeted improvements. i-PARIHS could serve as a guide for planning future SSC implementation efforts, however, further clarification of facilitation processes would improve the framework’s utility.

**Trial registration:**

No health care intervention was performed.

**Supplementary Information:**

The online version contains supplementary material available at 10.1186/s12913-022-08680-1.

## Background

First developed and tested in 2008, the World Health Organization (WHO) Surgical Safety Checklist (SSC) has been adopted and implemented in over 130 countries in an effort to reduce surgical-related morbidity and mortality [1–3]. While the checklist has spread rapidly over the past decade, many institutions and professional societies of surgery and anesthesia adopted use of the checklist to improve surgical safety without fully understanding the effort it would take to implement it into daily practice [2]. This has resulted in varying degrees of successful checklist implementation into hospitals and surgical centers as well as its success in reducing surgical complications [1, 3–5].

SSC effectiveness has been limited in high-income settings given the universal nature of the checklist; implementation challenges [6, 7] stemming from perceived relevance and benefit of the checklist within the local context [8–10]; clinician resistance, especially among senior surgeons [11, 12]; hierarchy in the operating room (OR) [9, 11]; and, difficulties with integrating the checklist into existing workflows [8, 10]. The strategies selected to implement the checklist and the individuals who led this work were key factors in the extent to which these challenges impacted SSC implementation [8, 13–15]. For example, sites with clinician champions rather than health administrators faced less resistance from surgical team members [13, 14]. Conversely, sites that provided little education on the background of the checklist were less successful at illustrating its relevance and benefits to their staff [13].

Despite their growing popularity, healthcare quality improvement (QI) initiatives frequently result in few or no meaningful changes following their implementation [16]. The few that are successful tend to be difficult to sustain or their results are not able to be replicated in other contexts [16]. In addition, few published evaluations make any reference to a specific theory used in the development or implementation of health care quality initiatives, including the SSC [17–21]. A recent review of surgical quality and safety initiatives, focused around the SSC, concluded that underutilization of theories, frameworks, and models to guide the selection of implementation processes has hindered our ability to understand why this evidence-based intervention is successful in some contexts and not others. In order to accelerate improvements in the quality and safety of surgical care, it’s essential that developing evidence for implementation strategies is given the same importance as developing evidence for an intervention [21].

To address this, the current study explores the extent to which SSC implementation practices could be assessed, and gaps identified, through the constructs of an implementation framework. Given the complex, multi-disciplinary environments in operating rooms and surgical centers, we selected the integrated Promoting Action on Research Implementation in Health Services (i-PARIHS) framework. The i-PARIHS framework posits that successful implementation of evidence into health care practice stems from a combination of four key constructs: the characteristics of an innovation and quality of associated evidence (innovation construct); the influence of the recipients of the innovation and evidence (recipient construct); and aspects of the local, organizational, and external health system context into which the innovation is implemented (context construct) [22]. Integral to i-PARIHS is the idea that implementation is activated through a facilitation construct that evaluates and responds to the information identified through the innovation, recipients, and context constructs [22]. This facilitation concept appears to be essential in successful implementation of the SSC, and commonly identified barriers and facilitators to its implementation align well with the other three primary constructs. Previous studies have also found it effective in examining the implementation of quality interventions in similar fast-paced, multidisciplinary health care environments [23].

The aim of this study is to determine whether and how i-PARIHS could be used to help implementers develop structured and comprehensive implementation plans for the SSC in high-income hospitals and surgical centers. Further, while the parent PARIHS framework has been evaluated in several health care settings, there is still a lot to learn about how i-PARIHS can be used to analyze and prospectively guide implementation of evidence-based practices [23, 24]. This study examines the extent to which this framework can be used for interventions in high-income surgical environments and for developing targeted recommendations to improve SSC implementation efforts.

## Methods

### Study design and setting

This qualitative study supports a larger parent study aimed at developing a toolkit to facilitate SSC adaptation, implementation, and utilization in high-income settings [25]. The current study utilized interviews with surgical team members and health administrators to create a comprehensive understanding of the key elements necessary for successful implementation of the SSC. Interviews were conducted across five high-income countries: the United States (*n* = 9), Canada (*n* = 11), the United Kingdom (*n* = 7), Australia (*n* = 12), and New Zealand (*n*= 12). These countries were selected because of their similar resource availability, differing degrees of checklist utilization [25], and opportunity to examine how healthcare delivery systems may impact implementation and use of the SSC. Surgical team members included clinicians from surgery, nursing, and anesthesiology. All participants classified as nurses self-reported having a clinical role on the perioperative team. This study was reviewed and approved as exempt by the Boston University Medical Campus and Boston Medical Center Institutional Review Board (IRB#H-38776).

### Study participants

Interviewees were initially recruited through purposive sampling [26] of respondents to a survey conducted through the parent study that examined current attitudes toward SSC use in high-income countries [25] who indicated a willingness to participate in an interview. This was followed by non-probability discriminative snowball sampling [27]; interviewees directed us to other colleagues who may be interested in discussing their experiences with the SSC. Interviewees were selected to ensure representation of diverse levels of experience, practice setting, and reported attitudes toward the SSC across all five study countries. Table 1 provides a demographic overview of the interviewees. Interviewees were contacted via email explaining the study and inviting participation. Emails included a study information sheet and verbal informed consent was obtained before each interview. Interviews of each participant type continued until it was felt that no new information was being provided. Three authors (ME, JG, CL) reviewed the data and determined when saturation had been reached.

**Table 1:** Description of Participants (*n* = 51)

Gender	M = 20 (39.2%)
	F = 28 (54.9%)
	Prefer not to disclose = 3 (5.9%)
Country	Australia = 12 (23.5%)
	Canada = 11 (21.6%)
	New Zealand = 12 (23.5%)
	United Kingdom = 7 (13.7%)
	USA = 9 (17.6%)
Years in Profession	0–5 = 6 (11.8%)
	6–10 = 8 (15.7%)
	11–19 = 14 (27.5%)
	20 + = 23 (45.1%)
Job Role	Anesthesiologist = 15 (29.4%)
	Health Administrator = 8 (15.7%)
	Nurse = 13 (25.5%)
	Surgeon = 15 (29.4%)
Type of Healthcare Organization Where Participant Works	Acute Care Hospital = 35 (68.6%)
	Ambulatory Surgery Center = 4 (7.8%)
	Other = 2 (3.9%)
	Unknown = 10 (19.6%)
# of Beds in Healthcare Organization Where Participant Works	< 200 beds = 5 (9.8%)
	≥ 200 beds = 21 (41.2%)
	Not applicable/Not provided = 25 (49%)

### Data collection

Semi-structured key-informant interviews were conducted between July 2019 and February 2020. An interview guide was developed for this study covering (1) current checklist processes, (2) checklist leaders, (3) checklist implementation barriers and facilitators, and (4) checklist effectiveness. The interview guide developed for this study is provided in Additional File 1. The interview guide was reviewed by the study team iteratively to eliminate redundancy between questions and refine wording. The interview guide was pilot tested with field-based specialists and people in the target demographic to ensure 1) interpretability and understanding of questions, 2) questions were eliciting the intended information, and 3) interviews could be completed in approximately 60 min. Interviews were conducted by two trained members of the study team. Interviews were conducted in-person, remotely using Zoom, which allows for video and audio recording, or via phone if using Zoom was not possible. All interviews were audio-recorded and transcribed by an independent company [28].

### Analysis

All transcripts were inductively coded to allow codes to emerge from the data [29], and across-case analyses were conducted to identify related codes across multiple study countries and generate themes. Themes common across multiple study countries were then mapped to the 35 sub-constructs that comprise the four core i-PARIHS framework constructs listed in Table 2 [22]. To identify considerations for the different health care delivery systems in the five study countries that may impact SSC implementation guidance, we conducted within-case analyses [30] and mapped them to the External Health System Context sub-constructs of the i-PARIHS framework. All analyses were conducted using NVivo 12 (QSR International).

**Table 2:** i-PARIHS Innovation, Recipients, and Context Constructs and Sub-Constructs

Innovation	Recipients	Context
•Underlying knowledge sources •Clarity •Degree of fit with existing practice and values •Usability •Relative advantage •Trialability •Observable results	•Motivation •Values and beliefs •Goals •Skills and knowledge •Time, resources, support •Local opinion leaders •Collaboration and teamwork •Existing networks •Power and authority •Presence of boundaries	*Local Level:* •Formal and informal leadership support •Culture •Past experience with innovation and change •Mechanisms for embedding change •Evaluation and feedback processes •Learning environment *Organizational Level:* •Organizational priorities •Senior leadership and management support •Culture •Structure and systems •History of innovation and change •Absorptive capacity •Learning networks *External Health System Level:* •Policy drivers and priorities •Incentives and mandates •Regulatory frameworks •Environmental (in)stability •Inter-organizational networks and relationships

## Results

**Fig. 1:**
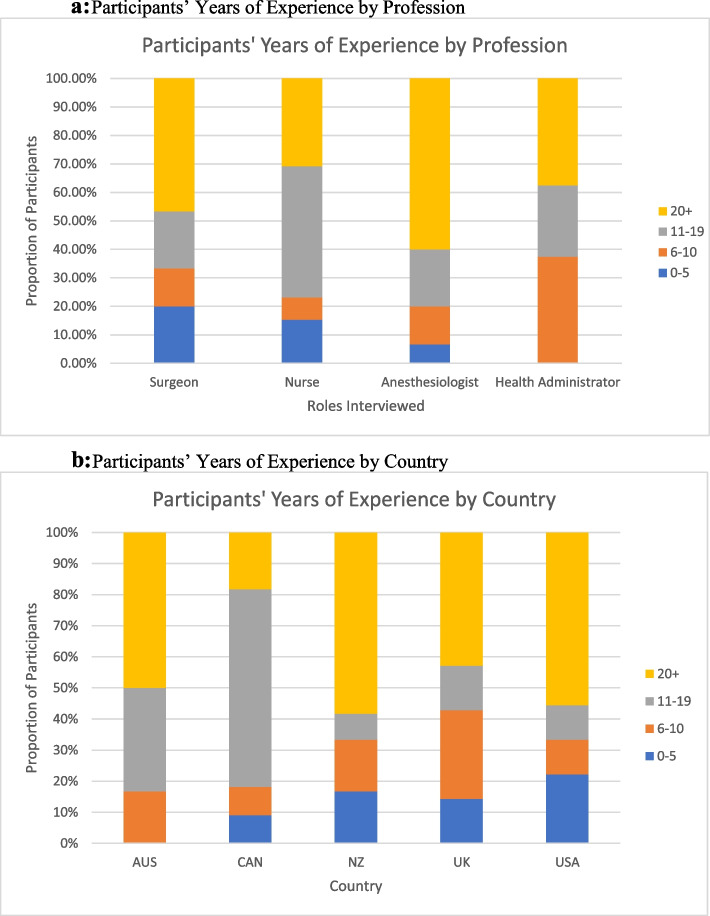
**a** Participants’ Years of Experience by Profession. **b** Participants’ Years of Experience by Country

Fifty-one stakeholders representing different roles in the surgical setting were interviewed: anesthesiologists (*n* = 15), surgeons (*n* = 15), nurses (*n* = 13), and health administrators (*n* = 8). Of the 51 interviews, 17 resulted from snowball sampling, and 34 were recruited through the survey. Table 3 depicts the number and profession of interviewees by country. Figure 1 a and b display the number of years of experience by profession and by country, respectively. While data were coded to the sub-constructs of the i-PARIHS framework, there were several instances of overlap; we present themes organized by the four main constructs for ease of interpretation and provide exemplary quotes to support key findings. Table 4 provides a full summary of the constructs, definitions, and key findings.

**Table 3:** Count and Frequency of Participants by Country

*Total Interviews (n = 51)*	
**United States**	**9**
Surgeon	1 (11.1%)
Nurse	5 (55.6%)
Health Administrator	3 (33.3%)
**Canada**	**11**
Surgeon	2 (18.2%)
Anesthesiologist	1 (9.1%)
Nurse	5 (45.5%)
Health Administrator	3 (27.3%)
**United Kingdom**	**7**
Surgeon	3 (42.9%)
Anesthesiologist	3 (42.9%)
Health Administrator	1 (14.3%)
**Australia**	**12**
Surgeon	4 (33.3%)
Anesthesiologist	7 (58.3%)
Nurse	1 (8.3%)
**New Zealand**	**12**
Surgeon	5 (41.7%)
Anesthesiologist	4 (33.3%)
Nurse	2 (16.7%)
Health Administrator	1 (8.3%)

**Table 4:** Summary of Constructs and Exemplary Quotes

Construct	Definition	Exemplary Quotes
Innovation	How evidence for and aspects of the program, policy, or intervention impact its uptake	“I think it could have been helpful to have a bit more – a little bit more formal training on it. Like maybe just – maybe more of the history, I would say, the background where it comes – and why it was introduced, why it’s thought to be important. And I think that helps, actually, people value it or get better buy-in to it.”—Anesthesiologist, CAN “They think it’s as a replacement for good systems in general. It’s not. It’s that final check.”—Surgeon, NZ “Well, the ultimate measure is lack of preventable adverse events. So, you know, anesthetizing a patient and then finding the prosthesis or a piece of equipment is not available means the checklist has failed. Certainly, wrong-site block or wrong-site surgery means that the checklist has failed, so a measure is the lack of adverse events.”—Anesthesiologist, AUS “And to my knowledge I can only remember one case where anything was significantly picked up during the checklist. I remember one day we had a patient ready for a knee scope and he was all like in the positioning devices and stuff. And we said, “Okay is this Mr. Smith for a left knee scope” and we looked down and we had the right knee ready. So that was picked up on the checklist…”—Nurse, CAN “…we don’t have good data on surgical site infection and its relationship to the checklist because we don’t get good data on surgical site infection.”—Health Administrator, UK “That all those people talking to each other at the beginning. Whereas, you know, some of those surgical techs in the Navy, they’re brand-new in the Navy, young, much lower rank than say, the surgeons or anesthesia are. And to get them to speak up and say what they need to say about that patient’s care, is great.”—Nurse, USA “Oh, that's the other thing too in EMR we made it a hard stop, we can't close or finalize our chart if we didn't do the elements of the timeout, like if we didn't do the fire risk because there's an entire segment about fire risk and this is a CMS requirement. So, we cannot close our chart if we didn't do the fire risk, if we didn't do the timeout. So those hard stops help in implementation, making sure that everybody does it.”—Nurse, USA “…the logic behind just having something on the wall is that everyone stops … yes, it’s only done verbally and yes, there’s no, you know, documented … you know, documentation of every single point that’s been checked off, but people are actually engaged and actually listen.”—Surgeon, NZ
Recipient	The degree to which intended recipients, individually and collectively, influence the implementation of the innovation	“There's the resisters, the people that think — often surgeons, but people that think that the safety check list doesn't apply to them, and I just think that’s disrespectful of our patients. And when it was first introduced in 2009 or whatever, there were trainees in our department that would say oh, I don't need to do this. And I always found that disappointing that they would watch surgeons role modelling that sort of behaviour and think that that was acceptable.”—Surgeon, NZ “I just think if the hospital wanted to implement that or wanted to make that a priority that they should have gone through the surgeons first because the nursing staff are more than willing to do these things but the problem is we get resistance from the surgeons.”—Nurse, CAN “You see with the young-—I think in general there's pretty good uptake, especially with some of the younger guys, and it's a cultural change and it takes a while.”—Anesthesiologist, NZ “I must say we had a number of adverse events with senior surgeons in a certain subspecialty and that changed immediately the practice in that unit; suddenly you realized that actually yes, we should be doing this because there’s someone who’s had effectively the wrong-site surgery with a senior surgeon involved and then all of a sudden overnight almost the checklist was adopted in that unit.”—Anesthesiologist, AUS “… a few months after the publication we had a case of wrong kidney in a very well-publicized case in the UK where it went to the criminal courts and everyone got a little bit worried. And that was quite useful.”—Anesthesiologist, UK “So, the way that I felt was the best way to make sure that everyone participated and listened and everything was to have me do it, since I would be the one actually starting the surgery, that I was the one who could kind of make the whole room stop and do the timeout effectively to make sure we had done everything. Because I know, I guess the WHO checklist recommends that like a nurse does it or something like that, but I just, I just didn't see that that was working. Umm, let's put it that way.”—Surgeon, USA “My biggest concern when it was originally brought in was by giving the surgeons the overall control of the time out…there will be people who aren’t going to speak up now…And it’s still probably a slight concern of mine. That if you’ve got a surgeon who’s running through the time out really quickly, which does happen, and you’ve got a nursing student in the corner who’s seeing something going wrong they’re not going to speak up to that surgeon.”—Nurse, NZ “Yes, so one thing that we really—I think that's been a major step forward for us is we made it very clear that the anesthesiologist should lead the sign in, the surgeon should lead the time out, and the nurse should lead the sign out. And that's improved ownership and it's improved sort of engagement from the team.”—Anesthesiologist, NZ “I think it’s quite good the way we run it here where different teams take responsibilities for different aspects. It sort of encourages sort of the team spirit, the team responsibility. Also, shared leadership because we try to avoid being hierarchical. I think if there’s one person who’s leading the checklist, people think well, it’s all their responsibility. We become passive observers but actually, everybody has shared responsibilities for different aspects of it. So, I think there’s a lot to be said about shared leadership when it comes to aspects in safety. It shouldn’t just be on one person’s shoulders.”—Surgeon, UK
Context: Local	How the resources, structures, culture and leadership in the Operating Room support or discourage implementation of the innovation	“But surgeons and how they come across because of their position of leadership, their attitude to the safe list is very pervasive on the team and if they are dismissive then, you know, why should everybody else buy into it, do you know what I mean?”—Surgeon, NZ “I have a very, very powerful, strong, effective, sensible CEO at my hospital. And he takes no prisoners. And he recently threw out the biggest financial earner in the hospital because of a problem. And a lot of CEOs would not have done that And I think that's the other thing that it needs. It needs a proper management who will stand up to maintaining standards, as opposed to, well we need the money.”—Anesthesiologist, UK “Because I do think that the sign in, or amalgamating the sign in and the timeout has some risks involved with it. My impression also is that all over the happiness with the process is rather low, which I believe is for various reasons because there's been recurrent changes and it sounds like there's a bit of change fatigue in terms of the surgical checklist.”—Anesthesiologist, AUS “…probably 15 to 20 min of that pathway between leaving the ward and hitting the operating table is completion of checklists… I think the number of checklists and the number of points could be reduced.”—Surgeon, UK
Context: Organizational	How the resources, structures, culture, and leadership in the hospital or surgical center impact the implementation of the innovation	“That's always—the hospital is concerned about how quickly we're turning over these cases because that's what generates income for the hospital so it can stay open, right? So, a lot of pressure with time is put on that, the critical safeguard's checklist such that it plays a big role in the success of it.”—Nurse, USA “There’s always a theatre efficiency time pressure to get the job done and I feel that the time pressure often goes against the Surgical Safety Checklist and people instead of paying attention to the items, it turns into, ‘Ah we just need to tick all the boxes and then we can proceed’.”—Anesthesiologist, AUS “I don’t think you can look at the checklist in isolation without also addressing the safety culture. It’s a tool to help change safety culture but if it’s used on its own it won’t change anything; you have to look at the whole safety culture of the department and hospital in order to affect real change,”—Health Administrator, UK “I think it just has – it has to be a culturally, not just acceptable, but a culturally desirable practice. And I'm not sure that we've cultivated that yet. We've got champions that do it, but those champions don't have opportunity to interact with other surgeons.”—Health Administrator, CAN “…one of the things that made it not spread as fast as it could is a culture of let’s not talk about our mistakes, let’s not talk about that fact that we caught that patient from having the hernia instead of their teeth. Instead of sharing it as a wonderful example of a save.”—Anesthesiologist, NZ “It’s mandatory, like there’s no – it’s not an option and we, every month we get a report back to say if there isn’t any that are complete and we do an investigation and we talk to the team and we find out what the barriers are or what happened. But it’s mandatory, it’s not an option.”—Health Administrator, CAN “I'm sure there's a policy that we have to do it but no one enforces that. Like there's no way to evaluate how well we're doing the checklist. It's just a yes or no whether it was done in our charting…”—Nurse, CAN
Context: External Health System	How the resources, structures, culture, and policies of the broader health system impact the implementation of the innovation	“The problem is we’re very isolated, we each work, we’re in a private hospital, so everyone works independently. So, there's no departmental or group policy. Private medicine here is different to a University Hospital, where there's a rule, we’re just pretty well do what we want. And that's where it may fall down in our hospital, where I don't know what the guy next door is doing.”—Surgeon, AUS “The private system looks at surgeons as being the customer. So, the surgeon’s the customer and the customer brings their patient. So, it’s a different way of looking at it and therefore they’re here running their business on our premises. So, we’re not in a position necessarily to say doctor, will you stop and listen, please. We try. But it shouldn’t be driven by the nursing staff. I really believe that it works – it’s far more successful if it’s driven by the medical staff.”—Nurse, AUS
Facilitation Processes	The active process that utilizes both facilitators and facilitation processes to integrate elements of the other three constructs	“…our hospital CEO has been very visible in supporting the safety huddle that we do and through that the prioritization of safety.”—Health Administrator, UK “Our CEO does regular rounds of our hospital, so there’s leadership rounds, so certainly part of that is visiting the theatre and – So all it takes for his interest to know and ask about it…”—Surgeon, NZ “…you need to get buy-in from people in every area. So, if you have anaesthetists and surgeons and nurses and non-medical…you want all of them on board, you have to get a couple of champions from my point of view for all of those areas to push it through their colleagues…”—Health Administrator, UK “The feedback from the audits? Immediate feedback coaching, kind of, you know, during that audit. And then there’s the scorecard, you know, how well each team did. Because we used to be divided up like you were the ortho team. You were the neuro team and kind of competition type thing. Like how well did you do? You know, peer pressure. That can accomplish a lot.”—Nurse, USA “For us getting over the implementation it took feedback – how many of the theatres are actually doing a checklist and which theatres are not doing a checklist, and so on.”—Surgeon, NZ “And it’s a team of probably 20 people that are going to be drifting in and out the whole day but they’re all there at the beginning and we go through everyone’s names, what everyone does and then we go through each case and discuss the needs for all various equipment. That’s a really good huddle.”—Anesthesiologist, AUS “That’s the time that you specifically think, “Okay, for my third operation I’m going to need – oh yeah, I’m going to need something different. I’m going to need X.” Then the nurses have two operations beforehand to prepare the actual instrument you need…”—Surgeon, NZ

### Innovation

The innovation construct identifies how evidence for and aspects of the program, policy, or intervention (here, the SSC) impacts uptake in different settings [22]. Sixty-five percent (*n* = 33) of respondents reported receiving education and training specific to the checklist at their institution. Most education seemed to focus on the logistics of the checklist processes, such as who participated and the checklist items included in each SSC phase. Interviewees felt that incorporating additional historical checklist information, greater clarity on its purpose, and the rationale for including different elements into the education and training would improve the SSC’s perceived relevance to their context and, in turn, buy-in from surgical team members. Interviewees stated:

*“…it could have been helpful to have…a little bit more formal training on it [the checklist]…more of the history, the background where it comes – and why it was introduced, why it’s thought to be important.” (Anesthesiologist, CAN)*

*“It’s quite powerful to know that if you give the antibiotics at the right time that it has a massive impact on perioperative infection. But if people don’t understand that then they’re not going to be engaged with that question.” (Anesthesiologist, UK)*

Interviewees discussed observable results from SSC use most frequently related to patient safety outcomes and adherence to evidence-based practices (EBP) rather than clinical outcomes frequently cited in literature, such as surgical-site infections reductions. Several comments pertained to the limited concrete examples of when the checklist was effective due to lack of clear data and the initial low rate of surgical-related morbidity and mortality in high-income settings. One interviewee mentioned:

*“…I can only remember one case where anything was significantly picked up during the checklist. I remember one day we had a patient ready for a knee scope …and we said, “Okay is this Mr. Smith for a left knee scope” and we looked down and we had the right knee ready. So that was picked up on the checklist…” (Nurse, CAN)*

Interviewees also commented on SSC usability and the format in which it was deployed throughout an organization. A combination of paper, electronic, and poster versions of the checklist were described. Electronic checklists, integrated into the electronic medical record (EMR), were cited most often by interviewees in the US and Australia, whereas the poster format was most frequently used in New Zealand. Interviewees who used electronic checklists liked that they could incorporate “hard stops” in the process that would prevent the surgical team from closing out a chart before key parts of the checklist were completed. For example:

*“…in EMR we made it a hard stop, we can't close or finalize our chart if we didn't do the elements of the timeout…those hard stops help in implementation, making sure that everybody does it.” (Nurse, USA)*

Those who used both the electronic and paper formats of the checklist commented on the ability to document checklist completion, which helped auditing compliance. However, those who utilized the poster format as an OR discussion guide discussed improved surgical team engagement with the processes compared to paper or electronic versions:

*“…the logic behind just having something on the wall is that everyone stops ... yes, it’s only done verbally and yes, there’s no…documentation of every single point that’s been checked off, but people are actually engaged and actually listen.” (Surgeon, NZ)*

A lack of education focusing on the background and rationale of the checklist and how it applied to a specific context hindered surgical team checklist acceptance. There was also a disconnect between the literature’s focus on the checklist’s improved clinical outcomes and the interviewees’ focus on patient-safety-related observable results from checklist use. Finally, the checklist’s format (e.g., electronic or poster) offered different advantages, with electronic checklists ensuring processes were completed, while poster-based checklists fostered more engagement with processes.

### Recipients

This construct indicates the degree to which people, individually or as teams, influence the implementation of the innovation [22]. Surgical team members, the recipients for the SSC, spoke at length about the degree of buy-in and processes for the checklist among SSC recipients. The overwhelming majority of interviewees (84%, *n* = 43) indicated that there was resistance and a lack of motivation to change existing practices among local opinion leaders, such as senior clinicians, related to the perception that the SSC was not relevant to them: *“When the checklist first came out, there was a lot of umming and ahing in the UK because most of us saw that we didn't have the dreadful number of problems…that were reported in the original paper,” (Anesthesiologist, UK)*. Half of interviewees (51%, *n* = 26) described how use of local, real-world examples of benefit are an effective way of improving SSC credibility among surgical team members. Examples of senior clinicians making mistakes after not using the checklist could be especially powerful:

*“We had a number of adverse events with senior surgeons in a certain subspecialty and that changed immediately the practice in that unit…there’s someone who’s had effectively the wrong-site surgery with a senior surgeon involved and then… overnight almost the checklist was adopted in that unit.” (Anesthesiologist, AUS)*

Contrary to the resistance seen from senior clinicians, several interviewees did report high levels of SSC acceptance among younger clinicians. These interviewees suggested continuing to work with this demographic to achieve sustained culture change. One Canadian nurse stated, *“…I find the younger ones are very focused on it and they're grabbing the checklist and they want to do it correctly, while some of the older surgeons, not so much.”*

In addition to recipient buy-in, interviewees discussed power dynamics between different medical roles impacting implementation success. Interviewees reported more resistance to and less compliance with SSC processes that were led by nurses instead of physicians. This was consistent across all five study countries. One interviewee said:

*“…it’s so they participate. Because if they’re just the nurse…they don’t pay attention. They don’t participate, they don’t do anything and to be quite honest, most of them think it’s stupid anyway.” (Nurse, CAN)*

Although physician-led checklist processes were associated with increased buy-in and compliance, interviewees expressed concerns that having surgeon-led processes would prohibit other team members from raising concerns. For example:

*“My biggest concern when it was originally brought in was by giving the surgeons the overall control of the time out…there will be people who aren’t going to speak up now… if you’ve got a surgeon who’s running through the time out really quickly, which does happen, and you’ve got a nursing student in the corner who’s seeing something going wrong they’re not going to speak up to that surgeon.” (Nurse, NZ)*

Some interviewees described how their organizations addressed this concern through the implementation of a shared leadership model for the checklist. For example, in New Zealand, anesthesiologists, surgeons, and nurses all took ownership over one phase of the checklist. This was reported to increase buy-in to and engagement with checklist processes from everyone in the OR:

*“… we made it very clear that the anesthesiologist should lead the sign in, the surgeon should lead the time out, and the nurse should lead the sign out. And that's improved ownership and it's improved sort of engagement from the team.” (Anesthesiologist, NZ*)

Existing surgical team hierarchies and resistance from local opinion leaders were critical barriers to successful SSC implementation. However, novel leadership strategies and emphasizing the SSC and other patient safety initiatives earlier in clinician training has resulted in SSC use becoming an expectation for newly trained clinicians.

### Context

#### Local level

The context construct identifies how the resources, structures, culture, and leadership, at the local, organizational, and external systems levels, support or discourage implementation of an innovation [22]. Local level is defined here as the context and physical environment within the OR. Critical to the success of SSC implementation was formal and informal leadership support at the local level. Formal leadership was defined as those who have authority in an organization, and informal leadership was defined as those with a high level of influence, but not necessarily authority, in an organization. OR leadership tends to match surgical team leadership, accordingly there were several concepts that overlapped with the recipient construct. In specifically examining the OR context, 20% (*n* = 10) of interviewees discussed the importance of formal OR leaders demonstrating checklist buy-in and leading by example establishing the OR culture. For example: *“…surgeons…how they come across because of their position of leadership, their attitude to the safe list is very pervasive on the team and if they are dismissive then why should everybody else buy into it?” (Surgeon, NZ)*.

Surgical teams’ past experiences with checklists also appeared to impact SSC implementation success. Interviewees from four of the five study countries mentioned concerns related to checklist or change fatigue. They noted both the number of checklists in use and the length of some of the modified SSC’s used in their organization as examples. Interviewees stated:

*“My impression also is that… happiness with the process is rather low, … because there's been recurrent changes…there's a bit of change fatigue in terms of the surgical checklist.” (Anesthesiologist, AUS)*

*“…probably 15 to 20 minutes of that pathway between leaving the ward and hitting the operating table is completion of checklists… I think the number of checklists and the number of points could be reduced.” (Surgeon, UK)*

Formal and informal leadership in the OR continued to be a critical factor in surgical team buy-in and engagement with the SSC. Surgical teams’ previous experiences with multiple, lengthy checklists in their workflows made them hesitant to participate in yet another checklist before starting a procedure.

#### Organizational level

At the organizational level, defined here as the hospital or surgical center, interviewees described a disconnect between the patient safety goals associated with the SSC and perceived organizational priorities. This resulted in less fidelity to checklist processes. Approximately 25% (*n* = 13) of participants noted time pressures as a key barrier to improved checklist implementation and use. Interviewees described pressure to complete surgical cases quickly and OR preparation for the next case as key barriers for checklist use. Time pressures resulted in surgical teams conducting an abbreviated version of the checklist or turning it into a “tick-box exercise” without engaging with the process:

*“There’s always a theatre efficiency time pressure to get the job done and I feel that the time pressure often goes against the Surgical Safety Checklist and people instead of paying attention to the items, it turns into, ‘Ah we just need to tick all the boxes and then we can proceed.’” (Anesthesiologist, AUS)*

Similar to organizational priorities, interviewees felt that having a strong organizational safety culture was key for promoting effective checklist use. One interviewee stated,

*“I don’t think you can look at the checklist in isolation without also addressing the safety culture. It’s a tool to help change safety culture, but if it’s used on its own it won’t change anything; you have to look at the whole safety culture of the department and hospital in order to affect real change,” (Health Administrator, UK)*.

Interviewees specifically cited the need to shift away from traditional punitive culture around making mistakes to using them as learning opportunities as a key aspect of effective safety culture. In the current culture, participants noted that it could be difficult to identify and share real-world examples because they were not encouraged to discuss mistakes. For example:

*“…one of the things that made it not spread as fast as it could is a culture of let’s not talk about our mistakes, let’s not talk about that fact that we caught that patient from having the hernia instead of their teeth. Instead of sharing it as a wonderful example of a save.” (Anesthesiologist, NZ)*

Within an organization, key contextual barriers to successful checklist implementation include time pressures resulting from organizational priorities that are perceived to be at odds with SSC goals. Contextual implementation facilitators aligned with a strong safety culture.

#### Health system level

While the five countries in this study have similar availability of health resources and services, delivery varies significantly. Our within-case analysis by country revealed a key consideration for centralized health systems that utilized both public and private hospitals. Interviewees from both Australia and Canada discussed challenges associated with providers being public employees rather than employed by a specific institution. In this structure, surgeons and other clinicians practice in both public and private institutions. For private hospitals in these settings, the provider is seen as the customer who has the ability to refer a patient to one of several institutions in the area. This incentivizes private hospitals to cater to the desires of the provider, and makes enforcing organization-wide policies difficult. Interviewees explained:

*“…the only way we get patients into our hospital is by the patient being referred from their general practitioner to a surgeon and the surgeon has got operating rights at this hospital… they have operating rights at several hospitals. It won’t just be this one… So, the emphasis is on protecting the surgeon and that’s how it is in private hospital.” (Nurse, AUS)*

Overall, limited information collected during the interviews mapped to this construct, and we found that there was often overlap with the organizational context. While these results apply to policy development and enforcement at the organizational level, they are a product of the national health structure, which is best represented through the external health system construct.

### Facilitation

The i-PARIHS framework defines facilitation as an active process that utilizes both facilitators and facilitation processes [22]. Just under half (41%, *n* = 21) of interviewees discussed that clear clinical and hospital leadership support for the checklist was a key facilitator in successful SSC implementation. Hospital leadership demonstrated both SSC buy-in as well as a broader commitment to patient safety. This helped align the SSC with organizational goals as illustrated by:

*“I have a very, very powerful, strong, effective, sensible CEO at my hospital. And he takes no prisoners. And he recently threw out the biggest financial earner in the hospital because of a problem… It [SSC] needs proper management who will stand up to maintaining standards, as opposed to, well we need the money.” (Anesthesiologist, UK)*

In addition, multidisciplinary clinical leaders could demonstrate their support by serving as a dedicated checklist champion and by setting a good example through meaningful engagement with the SSC processes in the OR. Having multiple champions, at least one for surgeons, one for anesthesiologists, and one for nurses, was the most effective strategy for obtaining buy-in from the entire team:

*“…you need to get buy-in from people in every area. So, if you have anaesthetists and surgeons and nurses and non-medical…you want all of them on board, you have to get a couple of champions from my point of view for all of those areas to push it through their colleagues…” (Health Administrator, UK)*

Interviewees also felt that auditing checklist use, specifically providing feedback on performance, was a key facilitator in obtaining buy-in and identifying teams who needed additional support. Seventy-six percent (*n* = 39) of participants mentioned auditing checklist use in their facility. However, only 19 (37%) interviewees reported receiving feedback on their performance. Performance feedback offered a data-driven insight into checklist use and impact in an organization:

*“…looking into why we’re getting the results. We’re doing and sharing that with the frontline practitioners, so that we understand what impacts we have on the patient after they leave our department.” (Nurse, CAN)*

Finally, participants from all study countries discussed incorporating a huddle held before the start of the surgical list as a facilitator for better incorporation of the SSC into their workflow. Huddles were used to introduce surgical team members, review all cases for the day including any anticipated challenges and equipment needs, and provide brief education. This allowed surgical team members to prepare in advance, avoiding delays later in the day:

*“…it’s a team of probably 20 people that are going to be drifting in and out the whole day, but they’re all there at the beginning and we go through everyone’s names, what everyone does and then we go through each case and discuss the needs for all various equipment. That’s a really good huddle.” (Anesthesiologist, AUS)*

Interviewees highlighted several facilitators for successful SSC implementation. Institutions where hospital administrative and clinical leadership publicly demonstrated their commitment to the SSC were able to improve buy-in among front-line staff. Establishing mechanisms for feedback on checklist performance illustrated its impact to the organization and helped identify surgical teams in need of additional support. Finally, modifying checklist processes to better incorporate the SSC into existing workflows, such as a morning huddle, improved surgical teams’ implementation success and efficiency.

## Discussion

To help address the gap in evidence-based implementation strategies, this study uses the i-PARIHS framework to analyze SSC implementation practices across multiple, international high-income settings. We identified key themes that impacted SSC implementation in each of the four main constructs and several that span multiple constructs. Based on our results, recommendations are proposed to improve future implementation efforts and the utility of the i-PARIHS framework in these contexts.

Our organizational context findings revealed a perceived disconnect between the stated patient safety goals associated with the SSC and organizational priorities. Interviewees discussed time pressures that resulted in the checklist not being done or becoming a “tick box exercise.” Previous studies have found that SSC use was frequently believed to negatively impact OR efficiency, and that this was a barrier to fidelity to checklist processes [8, 9, 31]. Closely tied to this was the belief that the checklist did not provide enough added benefit to justify the loss of OR efficiency [9]. Subsequent studies found that use of the SSC did not actually negatively impact OR efficiency [32], but this perception continues to be a barrier frequently cited by surgical team members.

In this study, interviewees mentioned that the development of a full-team morning huddle helped integrate the SSC into existing workflows, and improved checklist use. Use of a daily huddle is encouraged by multiple QI organizations to review the previous day’s work, look ahead to flag safety concerns in the day’s surgical cases, and review the SSC [33, 34]. Interviewees appreciated the opportunity to address potential concerns and ensure correct equipment procurement in advance, making the day flow more efficiently. The SURgical PAtient Safety System (SURPASS) checklist developed in the Netherlands has also incorporated pre-operative huddles as part of their 90-item checklist that covers all stages of care between patient admission and discharge [35, 36]. Implementers should consider the incorporation of a daily huddle as part of a comprehensive SSC implementation strategy.

Similarly, an organizational culture of punishing mistakes in health care continues to clash with a culture of process-improvement by incentivizing clinicians to hide mistakes [37]. Interviewees illustrated this through comments on the challenges some organizations faced in identifying and promoting real-world examples of SSC effectiveness, which was felt to be critical to improve buy-in. This builds on previous studies that identified punitive cultures around mistakes as an ongoing weakness in the healthcare sector [38, 39]. Promoting mistakes as learning opportunities can help empower surgical team members to openly advocate for SSC use by discussing the near misses and adverse events that occurred when it was not utilized properly. There have been recent initiatives aimed at changing how mistakes are viewed in healthcare, particularly by junior physicians [40]. As younger providers also appear to be more accepting of the SSC, there may be an opportunity to build a non-punitive culture around mistakes into SSC education and training.

An examination of results mapped to local surgical team context revealed power dynamics between team members stem from long-standing surgical team hierarchies, impacting the level of engagement teams had with SSC processes. This is consistent with previous research identifying existing hierarchies as a barrier to successful implementation [9, 11]. While physician-led checklists had more participation across all surgical team members compared to nurse-led processes, interviewees voiced concerns that existing hierarchies would prevent team members from speaking up during a physician-led checklist. Multiple campaigns have been implemented to flatten existing hierarchies in surgical teams [41, 42], but long-term impacts of these interventions vary [43]. One approach implementers should consider, as mentioned by several interviewees, is to utilize a shared-leadership approach where each surgical team member is responsible for leading one part of the checklist. Those that were currently using this model emphasized its success at engaging and empowering all checklist participants in the OR. Supplemental educational initiatives aimed at improving non-technical skills, such as team communication and how to speak up in critical situations, should also be considered, but research has shown they are likely to be ineffective if used in isolation [43].

When examining data within the external health system context of each study country, we found that countries that employed surgical team members through the state or health region faced a significant challenge in enforcing checklist use in private vs public hospitals. While public hospitals could utilize more force in ensuring compliance with checklist procedures, private hospitals were incentivized to cater to physician preferences, and needed to focus more on strategies to improve the sense of relevance, buy-in, and ownership, rather than mandatory policies and consequences, to promote meaningful checklist use. However, as most physicians in these settings practice in both public and private institutions, a more effective approach is to address the underlying causes of non-compliance to provide consistent expectations around checklist use and ensure patient safety in all ORs. One way to do this is as part of a broader patient safety initiative, similar to the implementation of the SSC through the Scottish Patient Safety Programme (SPSP) [44]. This program utilized all local boards of health to implement and promote SSC use in their region while simultaneously working to change the culture around patient safety. In 2019, researchers found that the SPSP significantly reduced patient mortality and OR return rates across the country [44].

### Utilization of the i-PARIHS framework

We found i-PARIHS to be a helpful framework to organize and examine data pertaining to SSC implementation efforts. Analysis of our qualitative data through the i-PARIHS lens revealed gaps in current SSC implementation strategies that facilitate targeted improvements for future implementation and reimplementation efforts. Accordingly, this framework would serve as a comprehensive guide for structuring implementation approaches for the SSC and similar interventions in a high-income context. It could also be used as a guide for evaluating implementation efforts; other researchers have successfully used i-PARIHS for this purpose in health care settings [45].

Consistent with other studies utilizing i-PARIHS to analyze implementation data, we did find several areas of overlap between sub-constructs, making it difficult to exclusively divide data into a single construct [23]. For example, the local context subconstructs were inter-related with the recipients sub-constructs because surgical team members were both the targeted SSC recipients and serve as formal and informal leadership in the OR. While these areas of overlap did not impact our analyses, it is important for future implementers to note the inter-dependencies between constructs likely to result from this and consider the entire framework when creating a structured implementation plan.

Finally, while the facilitation construct is intended to serve as the key activation component for the innovation, recipient, and context constructs [22], there was little definition provided. The framework does include definitions for the essential characteristics of an effective facilitator, but examples of the types of processes facilitators would aid in utilization when designing their implementation strategies. Similar to the approach taken by other researchers [23], we decided to map activities interviewees felt facilitated successful SSC implementation in their institution to this construct so practitioners have an additional resource when designing SSC implementation plans or similar interventions.

We note several limitations. First, there is the potential for interviewer bias through confirmation bias that may affect the quality of data collected. To minimize this risk, both interviewers conducted the first four interviews together, providing feedback on the delivery of the questions immediately following the conclusion of each interview. Second, biased results are possible by having a single researcher perform the inductive coding and thematic analysis. To address this, study results were reviewed by multiple members of the research team to identify inconsistencies and gaps in the analyses. Third, both the sampling strategy and the nature of interview-based research limits the generalizability of study findings. To improve this, interviewee selection criteria was reviewed by several different research team members to minimize the effects of selection bias. We also included five countries to identify themes applicable across multiple high-income settings and better generalize findings, however, we were unable to recruit interviewees from all professions providing anesthesia in these countries (e.g. nurse anesthetists) which may bias the perspectives in the study. Finally, we do not claim to fully understand SSC implementation efforts in low-income settings. Further research is needed to explore those contexts.

## Conclusion

Despite widespread adoption and use, there is still significant variation in the implementation of the WHO SSC. This variation substantially contributes to the checklist’s effectiveness in high-income settings. Similar to most other QI initiatives in healthcare, few SSC implementation efforts report using a framework or model to structure their approach. The i-PARIHS framework enabled a comprehensive assessment of current implementation strategies, identifying key gaps and allowed for recommending targeted improvements. i-PARIHS could serve as a comprehensive guide for planning future SSC implementation efforts, however, further clarification of facilitation processes would improve the framework’s utility.

## Supplementary Information

**Additional file 1.** SSC i-PARIHS Interview Guide.

## Data Availability

The datasets used and/or analyzed during this study are available from the corresponding author upon reasonable request.

## Abbreviations

WHO: World Health Organization
SSC: Surgical Safety Checklist
OR: Operating Room
QI: Quality Improvement
i-PARIHS: Integrated Promoting Action on Research Implementation in Health Services
PARIHS: Promoting Action on Research Implementation in Health Services
EBP: Evidence-Based Practice
US: United States
UK: United Kingdom
CAN: Canada
AUS: Australia
NZ: New Zealand
